# The first Oligocene sea turtle (Pan-Cheloniidae) record of South America

**DOI:** 10.7717/peerj.4554

**Published:** 2018-03-23

**Authors:** Edwin Cadena, Juan Abella, Maria Gregori

**Affiliations:** 1 Escuela de Ciencias Geológicas e Ingeniería, Yachay Tech, San Miguel de Urcuquí, Imbabura, Ecuador; 2 Universidad Estatal de la Peninsula de Santa Elena, La Libertad, Santa Elena, Ecuador; 3 Institut Català de Paleontologia Miquel Crusafont, Universitat Autónoma de Barcelona, Barcelona, Spain

**Keywords:** Montañita/Olón, Paleobiogeography, Ecuador, Testudines

## Abstract

The evolution and occurrence of fossil sea turtles at the Pacific margin of South America is poorly known and restricted to Neogene (Miocene/Pliocene) findings from the Pisco Formation, Peru. Here we report and describe the first record of Oligocene (late Oligocene, ∼24 Ma) Pan-Cheloniidae sea turtle remains of South America. The fossil material corresponds to a single, isolated and well-preserved costal bone found at the Montañita/Olón locality, Santa Elena Province, Ecuador. Comparisons with other Oligocene and extant representatives allow us to confirm that belongs to a sea turtle characterized by: lack of lateral ossification, allowing the dorsal exposure of the distal end of ribs; dorsal surface of bone sculptured, changing from dense vermiculation at the vertebral scute region to anastomosing pattern of grooves at the most lateral portion of the costal. This fossil finding shows the high potential that the Ecuadorian Oligocene outcrops have in order to explore the evolution and paleobiogeography distribution of sea turtles by the time that the Pacific and the Atlantic oceans were connected via the Panama basin.

## Introduction

Sea turtles are iconic vertebrates that have inhabited Earth’s oceans for at least 125 Ma (See Cadena & Parham, 2015). However, their evolution and fossil record in South America during the Cenozoic (∼66 Ma to present) is still poorly explored and understood. At present, the South American fossil record of Cenozoic sea turtles (Chelonioidea: Dermochelyidae + Pan-Cheloniidae, following Parham & Pyenson (2010) is restricted to the Paleocene (Danian) *Pampaemys meridionalis* De la Fuente & Casadío (2000), from the Roca and Jagüel Formations, Northern Patagonia, Argentina, later changed to *Euclastes meridionalis* (Lynch & Parham, 2003; De la Fuente et al., 2009), and currently attributed as *Erquelinnesia meridionalis* Parham & Pyenson (2010); the Miocene *Pacifichelys urbinai*, represented by skulls, lower jaws, cervical vertebrae, a partial carapace and a few non-described plastron fragments, from the Pisco Formation, Department of Ica, Peru (Parham & Pyenson, 2010); and the dermochelyids: *Natemys peruvianus* also from the Pisco Formation, Peru (Wood et al., 1996), and undescribed remains from Chilcatay Formation (Oligocene to Miocene) of Peru (Brand et al., 2011).

**Figure 1:**
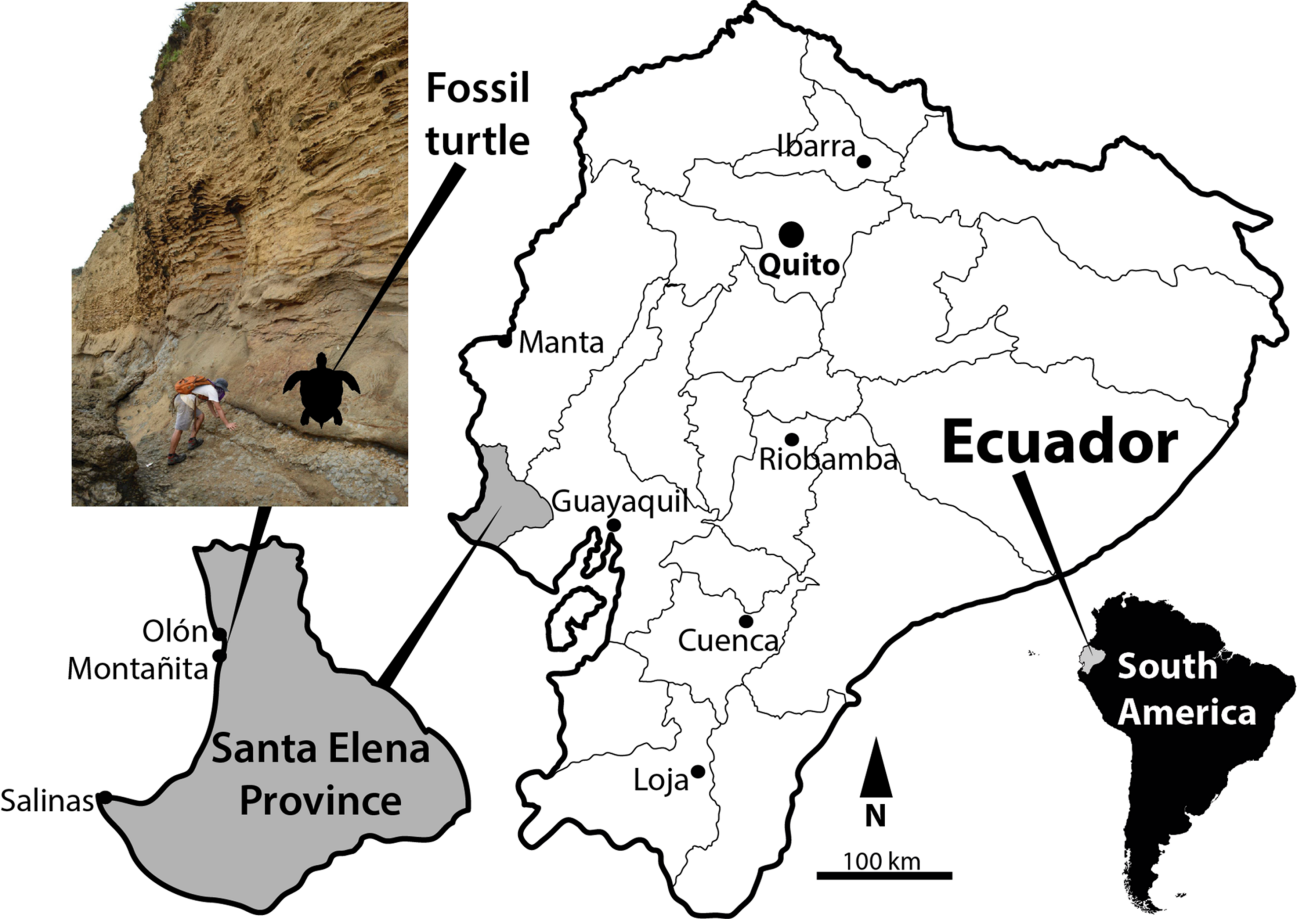
Map of Ecuador showing Santa Elena Province. Location and outcrop of Montañita/Olón locality from where UPSE-T0036 described herein was found.

Recently, a late Oligocene fossil site at the Pacific coast of Ecuador, Motañita-Olón locality (Fig. 1) has shown being rich in marine vertebrates, including a new genus and species of dolphin *Urkudelphis chawpipacha* (Tanaka et al., 2017), abundant sharks and actinopterygian fish teeth (J Carrillo-Briceño et al., 2018, unpublished data) and isolated turtle remains. Here we describe an isolated costal bone belonging to a sea turtle from this site, which constitute the first record of Oligocene Pan-Cheloniidae sea turtles in South America. In addition, we discuss the importance of this fossil site for understanding the evolution and paleobiogeography of sea turtles in the American continent.

## Materials and Methods

### Fossil material

The fossil costal bone described here is housed in the paleontological collection at the Universidad Estatal de la Peninsula de Santa Elena (UPSE), La Libertad, Santa Elena Province, Ecuador. Specimen UPSE-T0036. Comparisons of this fossil was done with some extant representatives of Cheloniidae as follow: *Caretta caretta* NMW 31531 and 1858; *Eretmochelys imbricata* NMW 1853 and MTKD D 8295; and *Lepidochelys olivacea* YT-Ver-0002. Permit for paleontological exploration of the Montañita/Olón locality was granted to J Abella by the Instituto Nacional de Patrimonio Cultural (INPC) of Ecuador, permit No  0039-DR5.INPC.2015.

## Systematic Paleontology

**Table:** 

**Figure 2:**
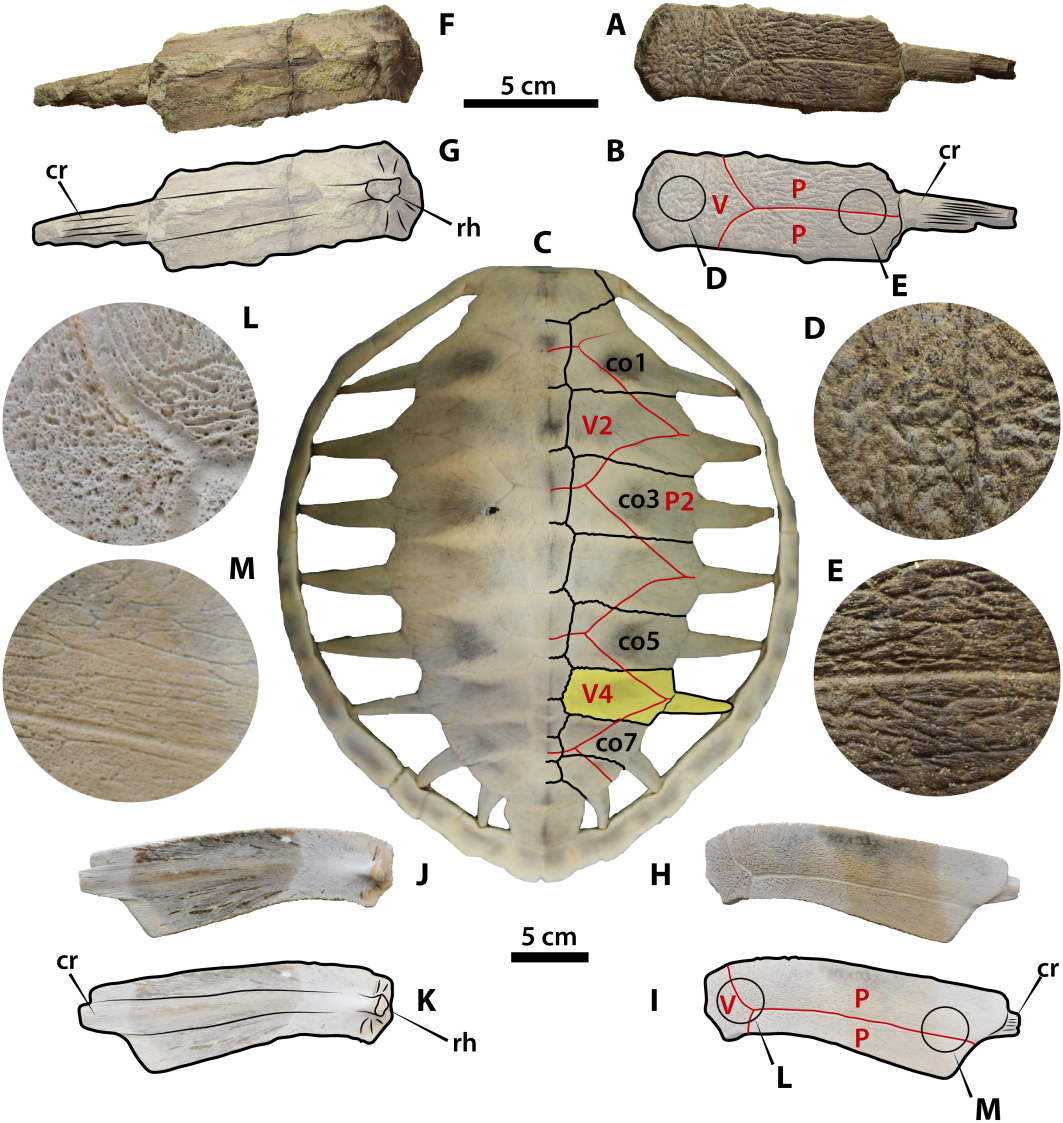
Pan-cheloniid (Gen. and Sp. Indt.) from Montañita/Olón locality compared with some extant marine turtles. (A–B). UPSE-T0036 right costal 6 in dorsal view. (C). Carapace of *Eretmochelys imbricata* MTKD D 8295, right costal 6 in yellow shadow. (D). Close-up of the medial region of UPSE-T0036 showing the pitted-vermiculated bone surface sculpturing (see circle (D) in (B)). (E). Close-up of the lateral region of UPSE-T0036 showing a bone surface sculpturing of anastomosing grooves (see circle (E) in (B)). (F–G). UPSE-T0036 right costal 6 in ventral view. (H–I). Right costal 6 of the extant *Lepidochelys olivacea* YT-Ver-0002 in dorsal view. (J–K). Right costal 6 of the extant *Lepidochelys olivacea* YT-Ver-0002 in ventral view. (L). Close-up of the medial region of *Lepidochelys olivacea* YT-Ver-0002 showing the pitted-vermiculated bone surface sculpturing (see circle (L) in (I)). M. Close-up of the lateral region of *Lepidochelys olivacea* YT-Ver-0002 showing a bone surface sculpturing of anastomosing grooves (see circle (M) in (I)). Top scale bar applies for (A–B) and (F–G), bottom scale bar applies for (H–I) and (J–K). Abbreviations: co, costal bone; cr, costal rib; P, pleural scute; rh, rib head; V, vertebral scute.

Testudines Batsch, 1788
Cryptodira Cope, 1868
Chelonioidea Baur, 1893
Pan-Cheloniidae Joyce, Parham & Gauthier, 2004
Gen. and Sp. Indet. (Fig. 2)

### Locality and age

Montañita/Olón locality, between the towns of Montañita and Olón, Santa Elena Province, Ecuador (1°48′50.64″S, −80°45′24.18″W). Here, we provisionally identify the source horizon for UPSE-T0036 as the Zapotal Member of the Dos Bocas Formation following Whittaker (1988). However, the age of this horizon is well constrained based on the occurrence of fossil shark *Carcharocles angustidens,* indicating that is it late Oligocene in age (Bristow, 1975; Tanaka et al., 2017).

### Description

UPSE-T0036 corresponds to a right costal 4 (14.5 cm length, 3.8 cm width as preserved) (Figs. 2A–2B). We use a specimen of the extant *Eretmochelys imbricata* MTKD D 8295 to indicate the anatomical position of UPSE-T0036 in a complete turtle carapace (Fig. 2C). UPSE-T0036 is a rectangular costal bone with almost the same medial and lateral regions width, lacking of fully ossified lateral region, which allows the exposure of the distal end of the costal rib. On its dorsal surface the bone exhibits a sculpturing pattern that varies along its width, being of dense vermiculation at the vertebral scute region (medial portion of the costal) (Fig. 2D), changing to anastomosing to almost parallel pattern of grooves at its lateral portion (Fig. 2E). The sulci between pleural and vertebral scutes are well defined, indicating that the vertebral scute covered 1/3 of the total surface of the bone, ending laterally in an acute tip. The sulcus between pleurals separates the bone in two almost equal portions. On its ventral surface (Figs. 2F–2G) the outline of the costal rib is defined along the length of the bone, showing a well-developed rib-head of the costal for the attachment with the thoracic vertebra.

## Discussion

### Taxonomical attribution and comparisons

UPSE-T0036 costal bone is attributed as belonging to Pan-Cheloniidae by sharing with some of the fossil and extant representatives of this clade the following characteristics: lack of lateral ossification but keeping a considerable thickness (5–7 mm), allowing the dorsal exposure of the distal end of ribs; dorsal surface of bone sculptured, changing from dense vermiculation at the vertebral scute region to anastomosing pattern of grooves at the most lateral portion of the costal. Lateral reduction in ossification of costals allowing the exposure of costal ribs occur also in some other turtles as for example Chelydridae (snapping and alligator turtles), however in these turtles the bone thickness is extremely reduced and the dorsal surface is smooth and developing ridges or knobs. Other groups of turtles that also exhibit reduction in lateral ossification of costals is the Tryionichidae (soft-shelled turtles), but in contrast to chelydrids and pan-cheloniids they develop a very distinct pitted dorsal bone sculpturing and absence of sulci from keratinous scutes.

Among pan-cheloniids UPSE-T0036 resembles the sculpturing pattern of other Cenozoic fossil forms from North and South America, as for example *Ashleychelys palmeri* Weems & Sanders (2014) from Charleston, South Carolina, USA, and the Miocene *Pacifichelys urbinai* Parham & Pyenson (2010) from Peru. However, it differs from the first one in having a narrower covering of the costal by the vertebral scute (as indicated by the sulcus). Unfortunately, the posterior region of the carapace is unknown for *P. urbinai*, avoiding to establishing if sculpturing pattern and scutes arrangement was similar as in UPSE-T0036. Other Oligocene sea turtles from South Carolina: *Procolpochelys charlestonensis* Weems & Sanders (2014) and *Carolinochelys wilsoni* Hay (1923) differ from UPSE-T0036 by having faintly sculptured to almost smooth dorsal carapacial bones. Table 1 shows the comparisons between UPSE-T0036 and Cenozoic taxa from American continent.

**Table 1:** Comparison of the morphological characteristics of costal bones of *Ashleychelys*, *Carolinochelys*, and *Procolpochelys*, with crown Cheloniidae (*Trachyaspis*, *Natator*, *Lepidochelys*, *Caretta*, *Chelonia*, *Eretmochelys*), with the addition of *Pacifichelys* Parham & Pyenson (2010) and UPSE T-0036 Pan-Cheloniidae (Gen. and Sp. Indet.) described herein. Table taken and modified from Weems & Sanders (2014).

Character	*Carolinochelys*	*Procopochelys*	*Ashleychelys*	UPSE-T-0036	*Pacifichelys*	Crown Cheloniidae
Costal bones surface texture	Sculptured and uniform along the entire bone surface	Faintly sculptured to smooth	Strong sculptured and uniform along the entire bone surface	Strong sculptured, pitted-vermiculate medially, anastomosing grooves laterally	Sculptured and uniform along the entire bone surface	Faintly to strong sculptured, uniform or with variation from the medial to the lateral portions of the bones.
Carapace thickness	Moderate	Thick	Moderate	Moderate	Moderate	Moderate
Vertebral scutes	Narrow	Narrow	Wide	Narrow	?	Narrow to wide

UPSE-T0036 resembles in geometry, sulci and medial to lateral sculpturing pattern variation of the posterior costals of some extant sea turtles, as for example *Lepidochelys olivacea* YT-Ver-0002 (Figs. 2H–2M), differing from this particular specimen by a wider covering of the vertebral scute on the costal surface. The width of vertebral scutes exhibit intraspecific variation as we observed in specimens of *Caretta caretta* NMW 31531 and 1858; and *Eretmochelys imbricata* NMW 1853 and MTKD D 8295 (Fig. 2C), for example in this last specimen the posterior vertebral scutes almost reach the most lateral portions of costal bones.

### Importance of Montañita-Olón locality for South American sea turtle evolution understanding

**Figure 3:**
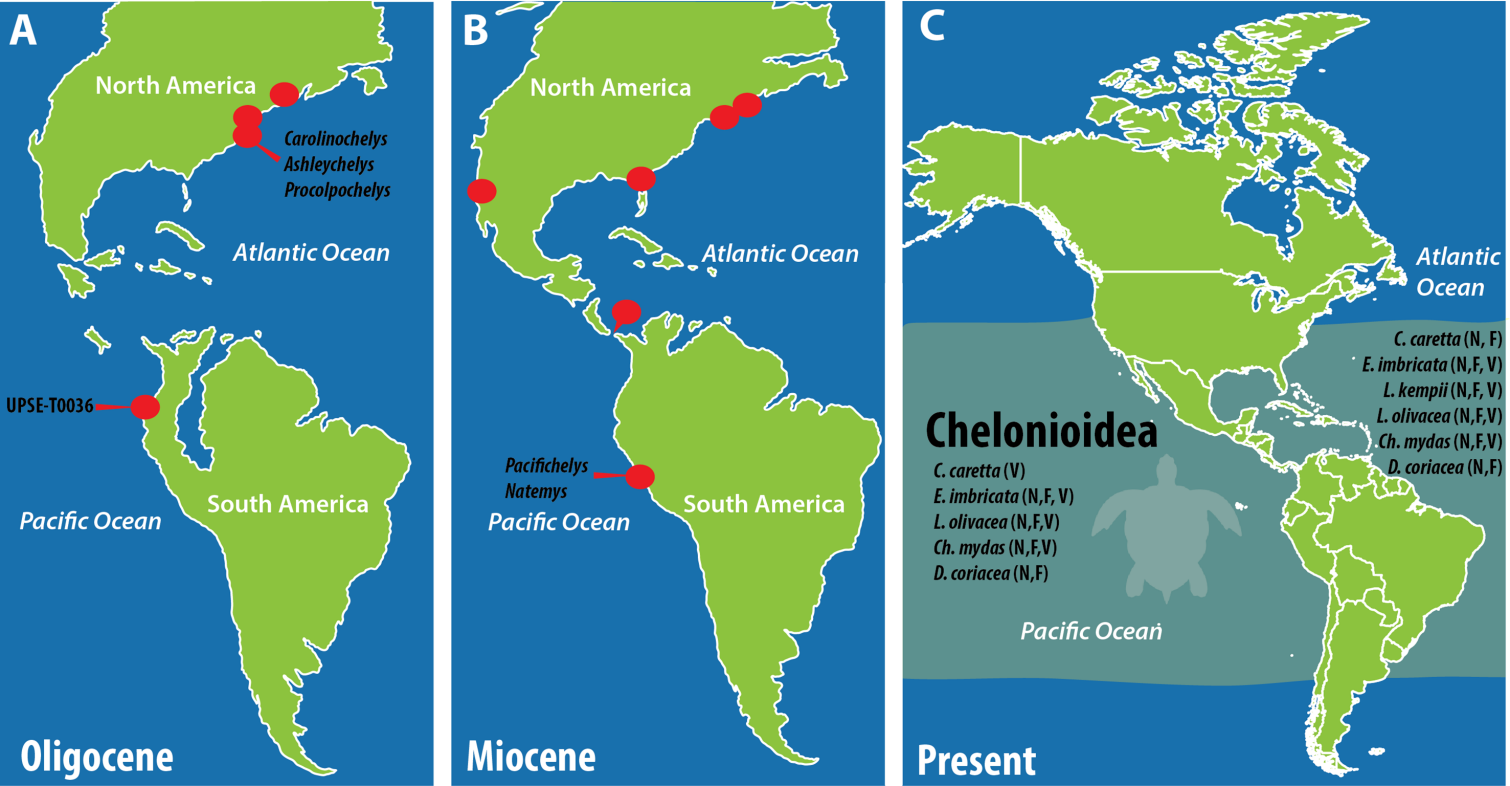
Paleogeographic reconstruction of South America and the fossil and extant distribution of chelonioidid marine turtles. (A) Oligocene occurrences including the first record of South America described here (UPSE-T0036). (B) Miocene occurrences including *Pacifichelys urbinai* from the Pisco Formation, Department of Ica, Peru (Parham & Pyenson, 2010); and the dermochelyid *Natemys peruvianus* also from the Pisco Formation, Peru (Wood et al., 1996) with the addition of the record from Panama basin (Cadena et al., 2012). (C) Present biogeographic distribution of marine turtles (Chelonioidea) based on Turtle Taxonomy Working Group et al. (2017). Red dots indicate fossil occurrences based on Fossilworks paleobiology database (Alroy, 2009) plus references mentioned above. Paleogeography taken and modified from Blakey (2016). Abbreviations: F, Foraging; N, Nesting; V, Vagrant.

The marine fossil vertebrates (cetaceans, sharks and turtles) recently discovered and described from the Oligocene, Montañita/Olón locality of Ecuador (Tanaka et al., 2017, J Carrillo-Briceño et al., 2018, unpublished data) represent the first occurrences of each of these groups in Paleogene (Oligocene) sequences of tropical South America; and for the particular case of turtles, the first Oligocene record of Pan-Cheloniidae marine turtles for the whole South America. Even though the material described herein corresponds to a single and isolated bone—reason why we avoid to formulate any further systematic or phylogenetic affinity hypotheses; it sets up a very promising scenario for future exploration and finding of new and more complete specimens that could elucidate if for instance the already known Oligocene sea turtle taxa from North America (Weems & Sanders, 2014; Weems & Sanders, 2017) inhabited also the tropical Pacific coast of South America; a hypothesis that it seems to be possible considering that during the Oligocene, the Pacific and the Atlantic oceans were connected via the Panama basin (Pindell, 1994; Boschman et al., 2014; Jaramillo, 2018) (Fig. 3A).

Thus, more complete sea turtle specimens from Montañita/Olón could shed light in establishing relationships with younger marine taxa from South America, as for example with the Miocene *Pacifichelys urbinai* Parham & Pyenson (2010) from Peru (Fig. 3B), or potentially being direct ancestors of any of the five extant representatives that inhabit the Pacific coast of tropical South America (Turtle Taxonomy Working Group et al., 2017) (Fig. 3C).

The fossil sea turtle material from Montañita/Olón also increases the knowledge on the fossil turtle paleobiodiversity of Ecuador, being the first record of a marine fossil turtle in the country and an addition to the already known occurrences of Pleistocene freshwater and terrestrial fossil turtles from Santa Elena Province (Cadena, Abella & Gregori, 2017).

## Conclusions

The costal bone descrided herein is the first undisputable record of Oligocene (late Oligocene, ∼24 Ma) marine turtles Pan-Cheloniidae of South America. This fossil finding shows the high potential that the Ecuadorian Oligocene outcrops have in order to explore the evolution and paleobiogeography distribution of sea turtles by the time that the Pacific and the Atlantic oceans were connected via the Panama basin. More complete specimens will have to be found in the Montañita/Olón in order to establish in detail the taxonomy and phylogenetic relationships of the Oligocene sea turtles that inhabited this part of South America. We hope this finding will encourage more paleontological expeditions and support for this type of studies in Ecuador and northern South America.

## Institutional abbreviations

MTKD: Senckenberg Museum of Natural History, Dresden collections, Germany.
NMW: Natural History Museum of Vienna, Austria.
UPSE: paleontological collection, Universidad Estatal de la Peninsula de Santa Elena La Libertad, Santa Elena Province, Ecuador.
YT: Yachay Tech paleontological collection, San Miguel de Urcuquí, Ecuador.

## Additional Information and Declarations

### Field Study Permissions

The following information was supplied relating to field study approvals (i.e., approving body and any reference numbers): The permit for paleontological exploration of the Montañita/Olón locality was granted to J Abella by the Instituto Nacional de Patrimonio Cultural (INPC) of Ecuador, permit No 0039-DR5.INPC.2015.

### Data Availability

The following information was supplied regarding data availability: Specimen UPSE-T0036 is stored at Universidad Estatal de la Peninsula de Santa Elena (UPSE), La Libertad, Santa Elena Province, Ecuador.
